# Novel Structural Approaches to Study GPCR Regulation

**DOI:** 10.3390/ijms18010027

**Published:** 2016-12-23

**Authors:** Marco A. Alfonzo-Méndez, Rocío Alcántara-Hernández, J. Adolfo García-Sáinz

**Affiliations:** Instituto de Fisiología Celular, Universidad Nacional Autónoma de México, Ciudad Universitaria, Ciudad de México 04510, Mexico; malfme@gmail.com (M.A.A.-M.); ralcanta@ifc.unam.mx (R.A.-H.)

**Keywords:** G protein-coupled receptors (GPCRs), posttranslational modifications, phosphorylation, ubiquitination, mass spectrometry (MS), GPR120, FFA4, α_1_-adrenoceptors, protein–protein interactions

## Abstract

Background: Upon natural agonist or pharmacological stimulation, G protein-coupled receptors (GPCRs) are subjected to posttranslational modifications, such as phosphorylation and ubiquitination. These posttranslational modifications allow protein–protein interactions that turn off and/or switch receptor signaling as well as trigger receptor internalization, recycling or degradation, among other responses. Characterization of these processes is essential to unravel the function and regulation of GPCR. Methods: In silico analysis and methods such as mass spectrometry have emerged as novel powerful tools. Both approaches have allowed proteomic studies to detect not only GPCR posttranslational modifications and receptor association with other signaling macromolecules but also to assess receptor conformational dynamics after ligand (agonist/antagonist) association. Results: this review aims to provide insights into some of these methodologies and to highlight how their use is enhancing our comprehension of GPCR function. We present an overview using data from different laboratories (including our own), particularly focusing on free fatty acid receptor 4 (FFA4) (previously known as GPR120) and α_1A_- and α_1D_-adrenergic receptors. From our perspective, these studies contribute to the understanding of GPCR regulation and will help to design better therapeutic agents.

## 1. Introduction

G protein-coupled receptors (GPCRs) are the largest family of membrane proteins, comprising as many as 3%–5% of the genes encoding proteins in sequenced genomes [1,2]. A hallmark of GPCRs is their ability to interact with a large variety of chemically diverse ligands. For this reason, GPCRs mediate key physiological processes, ranging from vision and olfaction to signaling in diverse organs and systems, such as the central nervous system, endocrine and immune networks, respiratory and digestive tracks, and many others. Thus, it is not an overstatement to say that this family of receptors participates in all of the major functions of vertebrates. This family of receptors is also involved in many human diseases, including heart failure, hypertension, diabetes, prostate cancer and bronchial asthma, to mention a few [3]. It is estimated that 30%–40% of drugs prescribed to treat these diseases target GPCRs [4] and, due to the large number of orphan receptors and tools for pharmacological screening, this number will probably increase [5]. It is currently estimated that approximately 800 human GPCR sequences exist, and they have been classified on the basis of their ligand-binding characteristics, signaling and sequence. Indeed, Fredriksson et al. [1] classified human GPCR sequences into five main families: Glutamate, Rhodopsin, Adhesion, Frizzled/taste2, and Secretin.

GPCRs are also denominated as seven transmembrane domain receptors due to their characteristic structure of an extracellular amino terminus, an intracellular carboxyl terminus and seven membrane-spanning segments connected by intra- and extracellular loops [6,7]. Seven transmembrane receptors usually transduce their signals by coupling to heterotrimeric G proteins that modulate the activity of enzymes (such as adenylyl cyclase or phospholipase C) and ion channels. These effectors increase or reduce the concentrations of second messengers (cyclic AMP, IP3, diacylglycerol, calcium, etc.) modulating enzymes, such as protein kinases, to further propagate signals within the cell. Furthermore, there is also evidence indicating that β-arrestins participate in GPCR signaling [4].

Upon activation, GPCRs undergo conformational changes that induce interactions between their intracellular domains and downstream signaling molecules, such as G protein subunits and β-arrestins [4,7,8]. GPCR posttranslational modifications (PTMs) include phosphorylation, palmitoylation, acetylation, glycosylation and ubiquitination, among others. Such covalent changes seem to play roles in the signaling and regulation of GPCRs [9,10,11,12]. GPCRs are frequently glycosylated at the amino terminus facing outside the cell, whereas the intracellular carboxyl tail is a substrate for phosphorylation, palmitoylation and ubiquitination. Among these PTMs, phosphorylation seems to be a key player in determining receptor desensitization/resensitization cycles [10], while ubiquitination is associated with lysosomal sorting and degradation [13,14].

The physiological or pathological outcome of GPCR-mediated signaling depends on the molecules with which the receptors interact. Actually, the PTMs mentioned above can induce tertiary and quaternary protein structural changes that regulate receptors association to other molecules and their function [15,16]. Therefore, to understand receptor signaling and regulation and to design rational GPCR-targeted drugs, it is necessary to characterize the conformational and structural receptor dynamics, as well as the specific GPCR interactome.

X-ray crystallography is the gold standard for investigating the structures of proteins and higher-order protein complexes at atomic resolution. After a gap from the first solved rhodopsin receptor crystal structure [17], 20 structures from the Rhodopsin family, two from the Secretin family, two from the Glutamate family and one Frizzled GPCR structure have been reported to date (see [18,19,20,21] and the references therein). The Nobel Prize in Chemistry was awarded to Robert Lefkowitz and Brian Kobilka in 2012 for their contribution to knowledge on the function and structure of β-adrenergic receptors [4,7].

Despite these great achievements, studies dealing with GPCR crystal structures remain challenging because it is very difficult to express and purify a sufficient quantity of any GPCR in an intact and functionally active form for direct experimental definition of its structural properties and binding interactions [22]. X-ray crystallography also has limitations, such as difficulties in monitoring protein dynamics [23]. Thus, there is only one crystal structure of a signaling complex available, i.e., the β_2_ adrenoceptor/Gs complex [24]. Although the crystal structure of β-arrestin-1 in complex with a phosphorylated V2 vasopressin receptor carboxyl-terminal peptide has already been reported [25], no crystal structure of a GPCR subjected to PTMs has been reported yet. This makes it hard to characterize the signaling structure/function mechanism of GPCRs. For these reasons, it seems necessary to complement studies on GPCR structures with post-translational modifications and associated molecules using other methods.

Mass spectrometry (MS) is useful not only for identifying protein sequences [25] but also for examining their structures after PTMs, folding and dynamics [26,27]. After important improvements on MS methodology, this technique has emerged as an important addition to X-ray crystallography and has been used in studies of protein structure and dynamics. Moreover, MS experiments require a small amount of sample, have no mass limits, allow rapid processing and can be used in high-throughput analysis [28,29,30]. The aim of this review is to present how MS-based studies are contributing to our knowledge on GPCR posttranslational modifications, associated proteins and receptor function/structure.

## 2. Posttranslational Modifications of G Protein-Coupled Receptors (GPCRs)

PTMs are a series of processes that can vary among cells, depending on the repertoire of proteins expressed, whose functional repercussion can also be affected by different cell stimuli and conditions, which affect protein abundance, trafficking, cell location and stability [31,32,33]. Currently, 469 different PTMs are reported in the UniProt database (http://www.uniprot.org/): 326 in eukaryotes, 250 in bacteria, 80 in archaea, and more than 100 in *Homo sapiens*. According to PhosphoSitePlus (http://www.phosphosite.org/) [34], protein phosphorylation is the most common PTM and has been detected in approximately 17,500 proteins of the human proteome. Frequent modifications include ubiquitination (approximately 8100 proteins), lysine acetylation (approximately 6700 proteins), lysine methylation (approximately 2400 proteins) and glycosylation (approximately 4500 proteins). The less frequently reported PTMs include succinylation, SUMOylation, citrullination, neddylation, disulfide bonding and lipidation [35]. Importantly, >95% of these data have been derived from MS-based proteome studies. Some advances on the most studied PTM, phosphorylation, with reference to a closely associated PTM (ubiquitination), are subsequently presented.

### 2.1. Phosphorylation

The phosphorylation state of a given protein is the result of the activity of two groups of enzymes: protein kinases and protein phosphatases. Protein kinases are phosphotransferases that transfer the γ phosphate group from ATP into serine, threonine or tyrosine amino acid residues, whereas phosphatases are hydrolases that release phosphate groups from those residues. Many GPCRs are subject to phosphorylation, and there is evidence suggesting that this PTM is associated with receptor desensitization and intracellular trafficking [9,32,36,37]. Furthermore, evidence also exists for phosphorylation-independent attenuation of signaling [38]. GPCR phosphorylation can take place at tyrosine residues (see for example [39,40]); however, serine/threonine phosphorylation is much more common and has been more extensively studied. This observation likely reflects that serine/threonine protein kinases are among the main modulators of these receptors. Currently, it is indicated that in homologous desensitization (agonist-dependent response-attenuation), G protein-coupled-receptor kinases (GRKs) are the major players. However, in heterologous desensitization (receptor activation-independent signaling-attenuation), second messenger-dependent protein kinases, such as protein kinase C and protein kinase A, and other protein kinases are the major players [10]. However, it seems to be an oversimplification to attribute all receptor phosphorylation that takes place during homologous desensitization to the action of GRKs; see, for example, the role of EGF transactivation in α_1B_-adrenoceptor phosphorylation induced by noradrenaline [41].

GPCR phosphorylation seems to be required for appropriate association with β-arrestins, and a phosphate sensor has been suggested in β-arrestin-1 on the basis of functional and crystallographic data [25]. It is interesting that multiple phosphorylation sites exist in most GPCRs and that they are mainly located at their carboxyl termini and third intracellular loops. Experimental evidence has shown that different phosphorylation patterns exist on GPCRs, depending on what ligand activates them and the cell in which they are expressed on; such “phosphorylation codes” can determine receptor function [31,32,37,42]. Different patterns of GPCR phosphorylation exist in a cell-specific manner also with different ligands (total agonist, partial agonist or inverse agonist), which can differentially regulate such phosphorylation patterns. Interestingly, this can lead to preferential signaling towards one action rather than others, a phenomenon known as biased stimulation [31,32,37,42]. The possibility that PTMs might determine the pharmacodynamic behavior of ligands is a current area of intense research.

As already indicated, GPCR phosphorylation at the carboxyl terminus and intracellular loops leads to β-arrestin association and receptor uncoupling from cognate G proteins. This represents a switch from G protein mediated- to β-arrestin-mediated-signaling. β-Arrestins modulate numerous pathways, primarily through recruitment of β-arrestin adaptor proteins [43]. These include the clathrin adaptor AP2, NSF (Nethylmaleimide-sensitive fusion protein, an ATPase involved in membrane fusion), ARF6 (ADP-ribosylation factor 6, a small G protein involved in vesicular traffic which participate in GPCR endocytosis) and also kinases of the mitogen activated protein kinase pathway [43].

Phosphorylation site prediction by in silico analysis is also a very useful tool. Advances in bioinformatics have allowed frequent confirmation of predictions by in cellulo experiments. The quantitative MS-based approach, with its high sensitivity and specificity, and mutational analysis are being increasingly applied in protein phosphorylation analysis, including phosphorylation sites in GPCRs, overcoming the limitations of conventional approaches, such as sequence motif analysis and site-directed mutagenesis [44,45]. Through tandem affinity purification and MS, the amino acid sequence and phosphorylation sites can to be determined simultaneously and unequivocally. Many GPCRs are known to be phosphoproteins. However, only in a limited amount of cases have agonist-induced and heterologous desensitization-associated GPCR phosphorylation been studied by MS in cellulo. This is rapidly changing since more groups are using this approach, and it is very likely that much more information will be available within a few years. A non-comprehensive list of GPCRs that have been studied using MS is presented in Table 1. In many cases, functional consequences have been suggested on the basis of a series of consistent evidences; in others, site-directed mutagenesis studies have been used to support such possibilities.

### 2.2. Ubiquitination

Ubiquitination is the second most frequently studied PTM in GPCRs. Ubiquitination of GPCRs and ubiquitination of adaptor proteins have been shown to regulate the GPCR endocytic pathway or GPCR trafficking [59,60]. Ubiquitin is a polypeptide of 76 amino acids residues (~8.5 kDa) that when attached to GPCRs promote receptor sorting into degradative pathways, typically on early to late endosomes or maturing vesicular bodies. Receptor proteolysis leads to a decrease in the total number of GPCRs available for signaling, a process known as “down-regulation”, which forms a part of long-term signaling attenuation (reviewed in [61], see also the references therein).

Ubiquitination is carried out by an enzymatic cascade involving the sequential activity of three ligases: E1, E2 and E3. Through their action, ubiquitin moieties are covalently and reversible attached to protein substrates, mainly on the ε-amino groups of internal lysines or, less frequently, on the free amino group at the amino-terminus of substrates [59]. E3 ubiquitin ligases have been shown to interact with GPCRs either directly through non-canonical WW-domain-mediated interactions or indirectly through interactions involving adaptor proteins. Many GPCRs seem to be degraded by metallo-proteinases present in lysosomes. However, before this take place, the ubiquitin moieties are removed by ubiquitin-specific peptidases (named USPs) ([61] and the references therein). Interestingly, removal of the GPCR ubiquitin moiety by peptidases is also involved in recycling GPCRs to the cell-surface for resensitization [61].

Ubiquitination of ~40 different GPCRs has been reported and among the best studied, is the β_2_-adrenoceptor. Agonist-activation of this receptor leads to very rapid phosphorylation, followed by ubiquitination; this latter PTM decreases hours later, correlating with receptor degradation [62]. Using MS, the ubiquitinated sites have been mapped at K^263^ and K^270^, in the third intracellular loop, and at K^348^, K^372^ and K^375^, in the carboxyl terminus [63]. β_2_-Adrenoceptors, in which lysines were mutated, internalize into endosomes upon agonist-activation but are not degraded in the lysosomes [63,64]. In contrast, β_2_-adrenoceptors in which phosphorylation sites were mutated exhibited impaired ubiquitination as well as reduced β-arrestin interaction [62]. Therefore, agonist-stimulated β_2_-adrenoceptor ubiquitination requires prior receptor phosphorylation and β-arrestin binding [62,65]. Interestingly, carvedilol, a β-adrenergic “antagonist”, frequently used in medical practice for the treatment of cardiovascular diseases, induces β_2_-adrenoceptor-dependent β-arrestin signaling, receptor ubiquitination, internalization, endosomal trafficking, and degradation [65]. The action of carvedilol seems to take place through a different molecular processes [66]. The pharmacodynamic classification of drugs acting on GPCRs is becoming much more complex (i.e., agonist/antagonist, inverse agonists/biased agonist/alosteric modulators) [67,68,69] but also opening new paths for therapeutic intervention.

A similar case exists for fingolimod, an S1P_1_ receptor (agonist but functional antagonist) that induces rapid receptor phosphorylation, ubiquitination, internalization and degradation [70]. Down-regulation of S1P_1_ receptor seems to play a key role in lymphocyte migration [71] and astrocyte activation [72]. These actions seem to explain why this agent has found a therapeutic niche in the treatment of multiple sclerosis [73].

An excellent comprehensive review on ubiquitination/deubiquitination of GPCRs was recently published and readers are referred to this [65].

## 3. Mass Spectrometry

MS is becoming a key technique to identify and properly characterize protein PTMs, including phosphorylation, ubiquitination, glycosylation, and proteolytic cleavage, among others. This highly specialized technique allows researchers to obtain the amino acid sequences that are modified. With this key information, bioinformatic analysis can be performed to obtain predictions on the structural consequences of such modifications as well as the putative enzymes responsible, and experiments can be designed to test the PTM functional consequences and relevance of putative participants in these processes. Practically, MS is very important because it can allow critical information to be obtained within a reasonable time frame and with high sensitivity [74,75].

MS requires three basic steps: (1) sample preparation, which includes obtaining a GPCR sample in sufficient quantity (usually to be detectable in Coomassie blue-stained gels) and of reasonable purity, as well as its proteolysis products under carefully controlled conditions; (2) sample ionization and detection, which involves sample bombing with electrons, fragmentation and ion formation, separation of the components according to their mass-to-charge ratio and detection by the mass spectrometer; and (3) analysis of sample data.

Researchers working in the GPCR field, including our group, are frequently only marginally familiar with MS techniques. Although MS systems are becoming more common, as a part of the equipment present in academic facilities, the spectrometer’s sensitivity and the expertise of the scientist in charge of it remain critical. In our case, it has been essential to interact with scientists who specialize in MS in academic service units, while the use of qualified commercial services is also an option.

Like essentially all techniques, MS offers advantages and disadvantages. As indicated, its main advantage is the possibility of processing samples and obtaining reliable data within reasonable time frames. However, its disadvantages include its limited sensitivity to proteases and difficulty of sample ionization. The presence of contaminants in this high-sensitive assay can lead to misleading results. Simple MS is not quantitative, but quantitative data can be obtained through the use of heavy and light isotopes during cell culture, called isotope-coded affinity tag labeling [76]. In the case of GPCRs, the highly hydrophobic nature of their transmembrane domains remains a challenge. During the past decade, MS analysis has gained interest for elucidating protein–protein and ligand–protein interactions, validating interactions by double hybrid assays, co-immunoprecipitation assays and Western blot analysis. These different techniques together with advances in confocal microscopy, FRET (Förster resonance energy transfer)/BRET (Bioluminescence resonance energy transfer) and image analysis have greatly contributed to identification of cellular complexes and “interactomes”.

## 4. Tales of Three GPCRs

In the following subsections, we present some examples of what these new technologies have taught us. In the first case study, we present some recent advances on free fatty acid receptor 4 including its regulation by phosphorylation, identification and characterization of its phosphorylation sites, and their possible functional relevance. In the second case study, findings on proteins that associate and/or co-purify with α_1A_- and α_1D_-adrenoceptors and their possible relevance are presented. In these two subsections, the main emphasis is made on aspects that have already been published by different groups, and we are incorporating some findings from our laboratory that have not yet been disclosed and might be of interest to other groups working with these of other GPCRs.

### 4.1. Free Fatty Acid Receptor 4 (FFA4) Phosphorylation Sites

Free fatty acids are important metabolic fuels by themselves and are constituents of storage lipids, such as triglycerides and membrane lipids, including phospholipids and sphingolipids. In addition to these well-known biochemical roles, they exert functions as natural agonists for some nuclear receptors [77] and for a family of GPCRs, comprising four members: FFA1-FFA4, with differential affinity for distinct fatty acids [78]. FFA4 (previously known as GPR120) was deorphanized in 2005 [79], and it is abundantly expressed in the intestine, where it induces GLP-1 (glucagon-like peptide-1) release into the circulation, which modulates insulin secretion and participates in glucose homeostasis. Additional studies showed that FFA4-deficient mice develop obesity, glucose intolerance and a fatty liver. Furthermore, a dysfunctional variant of this receptor is associated with obesity and other metabolic disturbances in humans [80]. In addition, FFA4 activation induces insulin sensitization and anti-inflammatory effects [81], as well as a variety of other actions in different organs and tissues [78,82]. Our group and others have observed that FFA4 is a phosphoprotein whose phosphorylation state is modulated by agonists and activation of protein kinase C [42,83,84,85,86]. Unsurprisingly, agonist-induced phosphorylation does not seem to be mainly mediated by protein kinase C, but rather by other kinases, likely GRKs [83,85]. Agonist- and protein kinase C-mediated FFA4 phosphorylation seem to be associated with receptor internalization [83,86]. Our group was working on determining the phosphorylation sites using MS when a very elegant paper, employing MS, reported the FFA4 phosphorylation sites located at the carboxyl terminus and their importance, together with some acidic residues (E^341^, D^348^, and D^355^), in the receptor association with β-arrestin-2 (also known as arrestin-3) [42]. Using a mutagenesis approach, other group also reported some FFA4 phosphorylation sites located at the FFA4 carboxyl terminus [85]. In Table 2, the identified phosphorylated residues and the techniques employed are presented. Phosphorylation site predictions were obtained using the Group Based Prediction System (GPS algorithm 2.1 v) [87,88]. Further work has shown that these residues alter different aspects of receptor function [86]; thus, mutation to alanine of S357 and S361 (named cluster 2) markedly altered receptor internalization and arrestin 3 recruitment, whereas similar mutations of T^347^, T^349^ and S^350^ (cluster 1) had no effect on receptor internalization and had minor effect on arrestin 3 recruitment, but markedly altered FFA4-mediated Akt activation [86]. Interestingly, in a recent review of this receptor, it was shown that these phosphorylation sites are conserved in human, rat and mouse FFA4 orthologs [89].

It is important to mention that truncation of the full C-terminal tail did not restrict activation of heterotrimeric G proteins [42], which allows molecular distinction between G protein-mediated and arrestin-mediated signaling. This raises the possibility of ligand development for selective activation of one of these signaling pathways over the other. This is important considering the physiological roles of this receptor in metabolic syndrome, diabetes, adipose tissue development and inflammation [80,81,82,90]. We also show in Table 2 that there are putative phosphorylation sites not only in the carboxyl terminus but also in the third intracellular loop as predicted using the GPS algorithm [87,88] and also several suggested in a previous study [89]. MS data obtained by our group indicated that T^242^ is a phosphorylation site. At this point, the functional significance of this site remains to be determined (work in progress in our laboratory).

### 4.2. α_1_-Adrenoceptor Associated Proteins

α_1_-Adrenoceptors belong to a three-member (α_1A_, α_1B_, and α_1D_ subtypes) subfamily of GPCRs that mediate the actions of adrenaline and noradrenaline [90]. This receptor subfamily participates in many physiological actions of catecholamines (regulation of blood pressure, urogenital functions, intermediary metabolism, among many others) and in the physiopathology of some diseases (hypertension, benign prostatic hyperplasia, among others) [91]. It is well-known that these adrenoceptors are phosphoproteins whose phosphorylation state is modulated by GRKs and protein kinase C and that their phosphorylation is associated with desensitization and internalization [9,92,93,94,95,96,97,98,99,100,101,102,103,104,105]. The elegant pioneering work of Cotecchia and coworkers identified, by site-directed mutagenesis, the GRK- and protein kinase C-target sites at the carboxyl terminus of α_1B_-adrenoceptors [99,100,101]. The phosphorylation sites in the other two α_1_-adrenoceptors have not yet been identified.

During our work on α_1A_-adrenoceptors using immunopurification and MS, we observed that an important number of proteins associate during the purification steps with this adrenoceptor, which were clearly and consistently observed in the different analyses performed. Some of these proteins are listed in Table 3. These include proteins involved in cell signaling, vesicular trafficking and degradation pathways, among others. It is clear that co-purification and detection by MS only suggests, but does not probe, direct or indirect (i.e., through the formation of mega-complexes or signalosomes) interactions with the GPCR of interest or play a role on signaling or regulation. However, published functional data are consistent with such possible interactions and might be provocative enough to be explored by different experimental approaches.

MS analysis suggested the possible interaction of α_1A_-adrenoceptors with enzymes and adaptor proteins previously reported as elements that participate in the regulation of this receptor subtype (phosphorylation/desensitization/internalization). This includes protein kinase C isoforms, such as protein kinase C α [106] and δ [107]. Surprisingly, the atypical isoform ζ was also detected. Other elements include subunits of phosphoinositide 3-kinase, which is known to participate in α_1A_-adrenoceptor phosphorylation and heterologous desensitization induced by okadaic acid and phorbol esters, as well as to co-immunoprecipitate with the adrenoceptor. Additionally, the regulatory subunit δ isoform of PP2A was also observed via MS of immuno-purified α_1A_-adrenoceptors.

Proteins, such as clathrin, dynamin, and Rabs, that participate in the internalization and trafficking of GPCRs have been identified as common members of GPCRs-complexes. In our MS studies, clathrin heavy chain, dynamin 2 and dynamin like-protein 1 were observed to be proteins that co-purify with α_1A_-adrenoceptors. It has be demonstrated that α_1A_-adrenoceptors co-localize with nuclear membrane protein lamina-associated protein 2 in adult cardiac myocytes, and it has been suggested to activate signaling at the nucleus [108] in our MS studies the presence of lamina 1 and lamina 2, exportins, and importins (Table 3).

Others proteins that were identified were members of the mitogen activated protein kinase family; myosin light-chain kinase; cytoskeletal proteins, such as actin, tubulin and myosin; and diverse elements of the degradation machinery, such as ubiquitin-protein ligase E3 and SUMO-activating enzyme subunit 2, among others (Table 3).

Differences in agonist-induced α_1A_-adrenoceptor phosphorylation, desensitization and internalization have been observed when the action of phenylethylamine agonists (such as phenylephrine and noradrenaline) were compared to imidazolines (such as oxymetazoline) [106]. The low-efficacy agonist, oxymetazoline, induces G protein-coupled receptor kinase-dependent α_1A_-adrenoceptor phosphorylation and it is followed by rapid desensitization and receptor internalization [106]. In contrast, phosphorylation of these receptors in response to noradrenaline largely depends on protein kinase C activity, it is not followed by clear desensitization, and the receptors undergo delayed internalization [106]. Loss of response after drug exposure is a particular problem for the vasoconstrictor effects of medications containing oxymetazoline. α_1A_-Adrenoceptor activation seems to play a key role in development of benign prostatic hypertrophy and treatment with selective antagonists seem to be of great help, ameliorating urinary symptoms [109]. Antagonists that could induce receptor internalization/down-regulation could be of potential therapeutic use. Understanding the receptor’s PTM that take place under the action of different agents, and their cellular consequences might be of help in developing of more effective drugs with fewer undesirable effects.

α_1D_-Adrenoceptors play a major role in the control of blood pressure and in the pathogenesis of hypertension [110,111,112,113]. It has been observed that this receptor subtype exhibits intrinsic activity of functional importance [96,114,115,116,117,118]. This subtype has been particularly difficult to study. When expressed, it exhibits a predominant intracellular location [119], which seems be due to a domain located at the amino terminus [120,121,122]; therefore, amino terminus truncation is a suitable experimental procedure to achieve α_1D_-adrenoceptor expression at the plasma membrane [96,114,120,121,122]. Interestingly, recent detailed work has shown, using receptor affinity purification and MS, that in multiple human cell lines, α_1D_-adrenoceptors are expressed both as the full-length form and also as an amino terminus-truncated protein [123]. A cleavage site was identified at the L910/V91 site, and it was suggested that the proteolytic processing of the amino terminus is a physiological mechanism to achieve membrane location of α_1D_-adrenoceptors with optimal functional properties [123].

Hague and coworkers [124,125] have shown that the α_1D_-adrenoceptor carboxyl terminus associates through a PDZ-interacting motif with syntrophins, which increases receptor expression and stability. The dystrophin proteins, syntrophin, dystrobrevin, and utrophin, were identified as α_1D_-adrenoceptor-interacting proteins [125]. MS analysis of purified α_1D_-adrenoceptors evidenced these and other associated proteins (Table 4). In our experiments using MS of immuno-purified α_1D_-adrenoceptors, a series of dynamin and many nuclear proteins were observed; however, in most cases, their possible roles in the receptor’s signaling and regulation are unknown (Table 4).

## 5. Perspectives

MS has emerged as a powerful complementary structural tool that complements information gained through X-ray crystallography and nuclear magnetic resonance, which both provide high resolution three-dimensional structural information for GPCRs and GPCR complexes. MS is a sensitive tool for identifying GPCR PTMs (phosphorylation and ubiquitination). It can be easily anticipated that the number of GPCRs whose PTMs will be determined in the very near future will markedly increase. These approaches together with the use of fluorescent confocal microscopy, FRET (Förster resonance energy transfer) and BRET (Bioluminescence resonance energy transfer) provide and will continue to provide information to gain a much deeper understanding of GPCR signaling and regulation.

Knowledge on the PTMs that affect specific receptor sites has already produced advances in our understanding of organ function. In a very elegant paper, Bradley and coworkers [126] reported a genetically engineered mouse expressing a G protein-biased M3-muscarinic acetylcholine mutant receptor, which allowed defining the role of receptor phosphorylation in bronchial smooth muscle contraction in health and in a model of asthma. G protein-dependent signaling and receptor phosphorylation-dependent signaling were mapped, which potentially predicts the outcome of biased agents [126]. Such findings and developments provide better approaches for drug design.

Ghrelin is a pleiotropic hormone secreted by the stomach that promotes food-seeking behaviors and a positive energy balance [127]. Studies on ghrelin receptors have also provided exciting insight on the functional relevance of GPCRs’ phosphorylation sites. Using MS the phosphorylation sites on the ghrelin receptor, GHSR1a, were defined as S^362^, S^363^ and T^366^; these residues are located at the carboxyl terminus and seem to be primarily responsible for β-arrestin binding [58]. Ghrelin receptor knockout animals did not show any clear change in body weight or energy consumption [128]. Interestingly, rats with a mutation deleting the distal part of the receptor’s carboxyl-terminal tail showed increased body weight and adiposity and reduced glucose tolerance [129]. Such mutation, that maintains GHSR cell surface abundance but alters its signaling properties, provided important insight into the role of the ghrelin receptor stressing the physiological role of the carboxyl terminus as a suppressor of ghrelin sensitivity [129].

Clearly, the previous two examples provide compelling evidence on the importance of carefully studying GPCR structure and function to better understand physiology and pathology, needed for developing tools for pharmacology and therapeutics.

## Figures and Tables

**Table 1:** G protein-coupled receptor (GPCR) studied using mass spectrometry. Amino acids in red indicate phosphorylation sites. Domain (DOM), carboxyl-terminus (C-term), third intracellular loop (3IL), references (Ref.).

GPCR	Main Phospho-Peptides Identified by MS	DOM	Functional Role	Ref.
Rhodopsin	DDDAS^334^ATASKTE	C-term	Inactivation	[46]
	DDDASATAS^338^KTE	C-term		
Rhodopsin	DDDAS^334^ATASKTE	C-term	Inactivation	[47]
	DDDASATAS^338^KTE	C-term		
	DDDASATASKTETS^343^QVAPA	C-term		
β_2_-Adrenergic	LPGT^384^EDFVGHQGT^393^VPS^396^DNIDS^401^QGRNCS^407^T^408^ND	C-term	In vitro phosphorylation. Desensitization	[48]
	LPGT^384^EDFVGHQGT^393^VPS^396^DNIDS^401^QGRNCS^407^T^408^ND	C-term		
	S^411^LLDLPGT^384^EDFVGHQGT^393^VPS^396^DNIDS^401^QGRNCS^407^T^408^ND	C-term		
β_2_-Adrenergic	FHVQNLS^246^QVEQDGRT	3IL	Desensitization	[49,50]
	RS^261^SKFCLKE	3IL		
	RSS^262^KFCLKE	3IL		
	AYGNYS^355^SNGNTGEQSGYHVEQEK	C-term		
	AYGNYSS^356^NGNTGEQSGYHVEQEK	C-term		
	LLCEDLPGTEDFVGHQGTVPSDNIDS^401^QGR	C-term		
V2-vasopressin	TGS^255^PGEGAHVSAAVAK	3IL	Not suggested	[51]
CXCR4	ALTSVSRGS^323^S^324^LKIL	C-term	Desensitization Internalization Signaling	[52]
Muscarinic M3	PS^384^SDNLQVPD	3IL	Signaling	[31]
	QAQKS^412^MDDR	3IL		
	QS^577^VIFHK	C-term		
Dopamine 2	HGLHSTPDS^321^PAKPEK	3IL	Desensitization Internalization	[53]
GPR120/FFA4	GAILT^347^DTS^350^VKR	C-term	Desensitization and Recruitment of arrestin 3	[42]
	GAILTDT^349^S^350^VKR	C-term		
	RNDLS^357^IISGYPYDVPDYA	C-term		
Apelin (APJ)	SAS^345^YSSGHSQGPGPNMGK	C-term	Biased signaling	[54]
	SASYSS^348^GHSQGPGPNMGK	C-term		
Neuropeptide FF2 (NPFF2)	AKS^369^HVLINT^375^S^376^NQLVQESTFQNPHGETLLYR	C-term	Desensitization	[55]
	KS^398^AEKPQQELVMEELK	C-term		
	ETTNSS^418^EIESAMVSK	C-term		
µ-Opioid	EFCIPTSSTIEQQNS^363^AR	C-term	Internalization	[44]
	EHPS^375^TANTVDR	C-term		
	QNT^370^REHPSTANTVDR	C-term		
κ-Opioid	RQS^356^T^357^NRVRNTVQDPASMRDVGGMNKPVTHHHHHR	C-term	Internalization	[56]
	QSTNRVRNT^363^VQDPAS^369^MRD	C-term		
Parathyroid hormone receptor 1 (PTHR1)	S^473^WSRWTLALDKR	C-term	Interaction with β-arrestins	[57]
	SGS^491^SSYSYGPMVSHTSVTNVGPR	C-term		
	SGSS^492^S^493^YSYGPMVSHTSVTNVGPR	C-term		
	SGSSSYSYGPMVSHT^503^S^504^VTNVGPR	C-term		
	VGLGLPLS^518^PR	C-term		
	PGTPALET^548^LETTPPAMAAPK	C-term		
	PGTPALETLETT^552^PPAMAAPK	C-term		
Growth hormone secretagogue receptor (GHSR1)	KLS^349^T^/350^LKDESSR	C-term	Endocytosis and recruitment of β-arrestins	[58]
	AWTES^362^SINT^366^	C-term		
	AWTESS^363^INT^366^	C-term		

**Table 2:** FFA4 receptor phosphorylation sites. Amino acids in red indicate phosphorylation sites; references (Ref.).

FFA4 Receptor Carboxyl Tail Sequence	Technique	Ref.
CRNEWKKIFCCFWFPEKGAILT^347^DT^349^S^350^VKRNDLS^357^IIS^360^G	In silico	[87,88]
CRNEWKKIFCCFWFPEKGAILT^347^DTS^350^VKRNDLS^357^IISG	Mutagenesis	[85]
CRNEWKKIFCCFWFPEKGAILT^347^DT^349^S^350^VKRNDLS^357^II SG	MS	[42]
CRNEWKKIFCCFWFPEKGAILT^347^DT^349^S^350^VKRNDLS^357^IIS^360^G	MS	[86]
CRNEWKKIFCCFWFPEKGAILTDTS^350^VKRNDLS^357^IIS^360^G	MS	Our data
**FFA4 Receptor Intracellular Loop 3 Sequence**	**Technique**	**Ref.**
S^226^YS^228^KILQITKAS^237^RKRLT^242^VSLAYSES^250^HQIRVS^256^QQDFRLFRT^265^LFL	In silico GPS	[87,88]
SYSKILQITKAS^237^RKRLT^242^VSLAYSESHQIRVS^256^QQDFRLFRTLFL	In silico	[89]
SYSKILQITKASRKRLT^242^VSLAYSEHQIRVSQQDFRLFRTLFL	MS	Our data

**Table 3:** Protein detected MS of immunopurified α_1A_-adrenoceptors. PKC, protein kinase C; PI3K, phosphoinositide 3-kinase; PP2A, protein phosphatase 2A; STAT, signal transducer and activator of transcription.

Detected Protein	Function	Possible Role
PKC α, δ and ζ	Serine/threonine protein kinase	Desensitization
PI3K	Phosphoinositide-dependent protein kinase	Desensitization
PP2A	Serine/threonine protein phosphatase	Resensitization
Dynamin 2	Scission of newly formed vesicles from de plasma membrane	Internalization
Clathrin	Formation of coated vesicles	Internalization
STAT1 and 3	Signal transducer and activator of transcription	Unknown
MAD2 and 4	TGF-β action, transcription factor	Unknown
Rab3	Membrane traffic	Vesicular traffic
Ubiquitin protein ligase E3	Ubiquitin ligase	Degradation
SUMO-activating enzyme subunit 2	E1-ligase for SUMO1/2/3	Degradation
Cullin-associated NEDD8-dissociated protein 1	E3 ubiquitin ligase complexes	Degradation
Exportin-1	Nuclear export of proteins	Unknown
Exportin-2	Nuclear export of proteins	Unknown
Importin-7	Prevents activation of Ran-GTPase	Unknown
Lamina B1	Nuclear structure and dynamics	Nuclear association
Lamina-associated polypeptide 2	Assembly of the nuclear lamina/nuclear organization	Nuclear association
Myosin	ATP-dependent motor protein	Unknown

**Table 4:** Protein detected MS of immuno-purified α_1D_-adrenoceptors. * Data from [125]; ** our data.

Protein	Function	Possible Role
* SNTB Syntrophin, β 1 and 2	Actin-binding protein	Adaptor protein
* UTRN Utrophin	Component of cytoskeleton	Adaptor protein
* ERLIN 2 ER lipid raft associated 2	Endoplasmic reticulum-associated degradation	Unknown
* GOPC	Golgi-associated PDZ and coiled-coil motif containing	Adaptor protein
* ERLIN 1	Endoplasmic reticulum-associated degradation	Unknown
* DTNA, Distrobrevin α	Cytoplasmic proteins bind to syntrophin	Adaptor protein
* SNTA1. Syntrophin α 1	Actin-binding protein, protein associated with dystrophin	Adaptor protein
* ACTA	Actin α 2 smooth muscle aorta	Cell motility
* AMFR	Autocrine motility factor receptor	Membrane traffic
* CKAP4	Cytoskeleton-associated protein	Cell motility
** OPA1	Dynamin-like 120 kDa protein, mitochondrial	Unknown
** DNM2	Dynamin-2	Scission of newly formed vesicles
** EEF2	Elongation factor 2	Unknown
** CSE1L, XPO1, XPO7	Exportin 1, 2 and 7	Unknown
** XRCC5	X-ray repair cross-complementing protein 5	Unknown
** MTHFD1	C-1-tetrahydrofolate synthase, cytoplasmic	Unknown
** UBA2	SUMO-activating enzyme subunit 2	Unknown
** NUP93	Nuclear pore complex protein Nup93	Unknown
** DDX21	Nucleolar RNA helicase 2	Unknown
** MCM7	DNA replication licensing factor MCM7	Unknown

## Abbreviations

GPCRs	G protein coupled-receptors
PTMs	Posttranslational modifications
MS	Mass spectrometry
GRKs	G protein-coupled receptor kinases
FRET	Förster resonance energy transfer
BRET	Bioluminescence resonance energy transfer
